# SG-ADVISER mtDNA: a web server for mitochondrial DNA annotation with data from 200 samples of a healthy aging cohort

**DOI:** 10.1186/s12859-017-1778-6

**Published:** 2017-08-18

**Authors:** Manuel Rueda, Ali Torkamani

**Affiliations:** The Scripps Translational Science Institute, Scripps Health, and The Scripps Research Institute, La Jolla, CA 92037 USA

**Keywords:** Mitochondrial DNA, Annotation, Healthy aging, Heteroplasmy

## Abstract

**Background:**

Whole genome and exome sequencing usually include reads containing mitochondrial DNA (mtDNA). Yet, state-of-the-art pipelines and services for human nuclear genome variant calling and annotation do not handle mitochondrial genome data appropriately. As a consequence, any researcher desiring to add mtDNA variant analysis to their investigations is forced to explore the literature for mtDNA pipelines, evaluate them, and implement their own instance of the desired tool. This task is far from trivial, and can be prohibitive for non-bioinformaticians.

**Results:**

We have developed SG-ADVISER mtDNA, a web server to facilitate the analysis and interpretation of mtDNA genomic data coming from next generation sequencing (NGS) experiments. The server was built in the context of our SG-ADVISER framework and on top of the MtoolBox platform (Calabrese et al., Bioinformatics 30(21):3115–3117, 2014), and includes most of its functionalities (i.e., assembly of mitochondrial genomes, heteroplasmic fractions, haplogroup assignment, functional and prioritization analysis of mitochondrial variants) as well as a back-end and a front-end interface. The server has been tested with unpublished data from 200 individuals of a healthy aging cohort (Erikson et al., Cell 165(4):1002–1011, 2016) and their data is made publicly available here along with a preliminary analysis of the variants. We observed that individuals over ~90 years old carried low levels of heteroplasmic variants in their genomes.

**Conclusions:**

SG-ADVISER mtDNA is a fast and functional tool that allows for variant calling and annotation of human mtDNA data coming from NGS experiments. The server was built with simplicity in mind, and builds on our own experience in interpreting mtDNA variants in the context of sudden death and rare diseases. Our objective is to provide an interface for non-bioinformaticians aiming to acquire (or contrast) mtDNA annotations via MToolBox. SG-ADVISER web server is freely available to all users at https://genomics.scripps.edu/mtdna.

**Electronic supplementary material:**

The online version of this article (doi:10.1186/s12859-017-1778-6) contains supplementary material, which is available to authorized users.

## Background

Next Generation Sequencing (NGS) technologies are revealing the complexity and richness of the human genome. While this revolution is blooming for nuclear DNA, much remains to be built out and matured for the 16,569 base pairs of the human mitochondrial genome (mtDNA), in particular for functional annotations of disease associated variants. The ability to more routinely analyze mtDNA samples is crucial to establishing a more robust description of the specific genetic variants underlying mitochondrial disease [1], considered in tandem with disease causative variants in the nuclear genome [2]. In that regard, the existence of heteroplasmy (the presence of multiple alleles in an individual) in mtDNA demonstrates that the mitochondrial genome is a rich source of *de-novo* mutations potentially underlying many rare conditions [3–9]. For deleterious mutations, a minimum critical proportion of mutated copies (in the range of 60%–90%) in the tissue(s) of relevance is necessary to display biochemical defects and phenotypic manifestation [4, 10]. The proportion of mutated copies (a.k.a. mutation load) can differ among tissues and it might not be detectable, may be harder to detect, or may not be representative of the mutational load in the tissue of relevance when ascertained in a single tissue homogenate or blood sample [11]. Thus, we envision that in the future an individual may be sequenced several times (at the tissue level) to develop a more accurate picture of the expected severity and tissue specificity of a suspected mitochondrial disease. For all these reasons, there is a need for robust bioinformatic analysis of mtDNA variants.

Currently, there are many free services available for non-bioinformaticians seeking to carry out variant calling of nuclear variants from whole exome (WES) or whole genome (WGS) sequencing, e.g. Galaxy [12], GenePattern [13] or WEP [14] among others. However, with the exception of the newly published server by Weissensteiner et al. [15], there are few (or no) options for services amenable to non-bioinformaticians that appropriately deal with mitochondrial data. Thus, when a researcher performs WES/WGS analysis producing a negative result, and would like to expand the analysis to the mitochondrial genome, he or she will need to perform an exploration of the Linux command-line tools (i.e., MToolBox [16], MitoSeek [17], mit-o-matic [18]; note that MitoBamAnnotator [19] is no longer available) and make a decision according to that search. Per our own experience, comparison of these tools is far from trivial and we believe it results in a barrier, especially for labs that do not have the willingness or the expertise, to systematically analyze mtDNA variants. This barrier not only arises from the non-user friendly nature of command line tools themselves, but also from the process required to install command line pipelines. It often happens that to implement a computational pipeline, especially from academic software, one needs to co-install a plethora of accessory components, mostly software-based, but some also hardware-based. For instance, to create the reference sequence *k-mers* needed to install Gmap [20] within MToolBox, one needs 32GB of RAM, which is double what a typical non-specialized workstation usually contains. In our case, after testing a repertoire of packages we selected MToolBox v1.0 due to its robustness and richness of results (a comparative review of MToolBox performance was published elsewhere [15]). MToolBox is a highly automated bioinformatics pipeline that includes mtDNA assembling from WES or WGS data [21], heteroplasmic fraction detection with a related confidence interval, variant call format (VCF4.0) output, haplogroup assignment [22] and variant prioritization according to a disease score [23]. MToolBox is indeed a powerful tool, but in terms of data visualization only has a basic GUI (MSeqDR; https://mseqdr.org/mtoolbox.php). For this reason in our laboratory we developed an alternative way of visualizing MToolBox results that we incorporated to the analysis of our cases from the Molecular Autopsy [24] and IDIOM [25] studies.

In this light, we present SG-ADVISER mtDNA, a web server built on top of MToolBox, that attempts to simplify the human mitochondrial DNA variant calling, annotation and interpretation of variants. SG-ADVISER mtDNA utilizes SAM/BAM files and uses dynamic HTML web tables to display the results. The server was built having simplicity in mind, and is built upon our own experience in interpreting mitochondrial DNA mutations in the context of sudden death and rare diseases. Along with the server, we also provide individual level results for 200 healthy aging individuals that we analyzed and compared to reference cohorts. Our objective is to provide a simple alternative for non-bioinformaticians aiming to acquire (or contrast) mtDNA annotations via MToolBox.

## Implementation

The SG-ADVISER mtDNA back-end was written in Perl 5. For the client-side operations, we used a responsive design web interface with HTML5 and JavaScript libraries. The entire core calculations are carried out by the MToolBox v1.0 suite as described elsewhere [16], as well as with in-house scripts (see Additional file 1: Text T1). The reference genome used is the Reconstructed Sapiens Reference Sequence (RSRS) [26].

### Input data

The server functions in two modes, “individual sample” and “cohort”. In the former, the user can upload a single SAM/BAM file, whereas in the latter the user can upload a whole directory consisting of multiple SAM or BAM files. Cohort mode is a good choice for family pedigrees or small-size populations, as the results for each variant will be shown aggregate in one line. We deliberately restricted the input to be SAM/BAM files, knowing that they have become a *de facto* standard for sharing sequence data. Rather than uploading the whole WES/WGS alignment file, we ask the user to upload only the mitochondrial DNA reads. This is the only “technical” step that needs to be performed prior to submission and can be easily achieved with SAMtools [27], as described on the help section of the server. This way, we avoid the unnecessary transfer of large data files over the network, much of which will not be processed anyway. Note that the server will re-align the reads with Gmap, so the alignment in the original file is just required to isolate reads mapping to the mtDNA genome. All the data transfer is performed securely through an SSL certificate. Uploaded SAM/BAM files are deleted after job completion and results are kept for a week, after that all the data are permanently deleted. Apart from the data upload, there are three optional parameters for the user: i) a text field for an email address to get notified when the job finishes, ii) an option to set the job to private so that only the user who sent the job will have the link to the results (note that an email is mandatory when this option is selected), and iii) a text field to change the default job identifier. Apart from a standalone calculation, we envision that some users may wish to use the Linux command line to launch multiple jobs. For that purpose, in the help section we provided scripts for web services that will avoid the necessity for “screen scraping” of HTML.

### Output data

Upon submission, each job is sent to a PBS queue system installed in a local dedicated server. The hardware consists of 1 x Intel Xeon CPU E5-2630 V4 2.2GHz with 64GB of RAM and 16 TB of HDD, capable of running 20 simultaneous threads. The alignment step with Gmap-gsnap [20] benefits from parallelization, thus, in a compromise between speed and capacity we set the number of threads per sample to 4. With this set-up, analysis of one sample at 2200X coverage takes ~2 min to complete. At full capacity the server will support ~150 samples per hour (~3600 samples per day). Once a job is submitted to the queue, the user is redirected to the status page that contains information about the completion of submitted jobs. When a job is finished, the results page becomes available via a link. In the results page, all the prioritized variants coming from MToolBox are displayed, as well as appended information that we extract from the final VCF files (i.e., heteroplasmic fraction, depth and genotype information). The HTML table has several functionalities, such as URL links to external databases, sorting, search (regular expressions allowed), rearranging of columns, etc. All the results can be downloaded as text files by clicking in the corresponding link in the page. The server includes a pre-computed example as well as the 200 individual Wellderly samples, plus a help page with extended documentation on the technical details.

## Results and discussion

### Analysis of the healthy aging cohort

The healthy aging cohort (a.k.a. the Wellderly) is defined as individuals who were > 80 years old with no chronic diseases and who were not taking chronic medications (see full criteria of inclusion at [28]). Here we analyzed unpublished data from 200 Wellderly individuals who had their WGS sequenced with the Illumina Moleculo technology [29]. For each individual, we extracted mtDNA reads from WGS BAM files to create mtDNA-only BAM files that were later submitted to our server. We set up the server so that all the data could be browsed and downloaded (see help page).

Apart from allowing visualization of the individual level data on the browser, given that mtDNA has been associated with aging in the literature [2, 6, 30–40], we carried out basic statistics on the abundance and distribution of variants within the mitochondrial genome. We would like to emphasize that our objective with this publication is not to perform a comprehensive case-control study, but rather to make the data publicly available along with the server.

### 1) Effect of depth of coverage on the number of variants

**Fig. 1:**
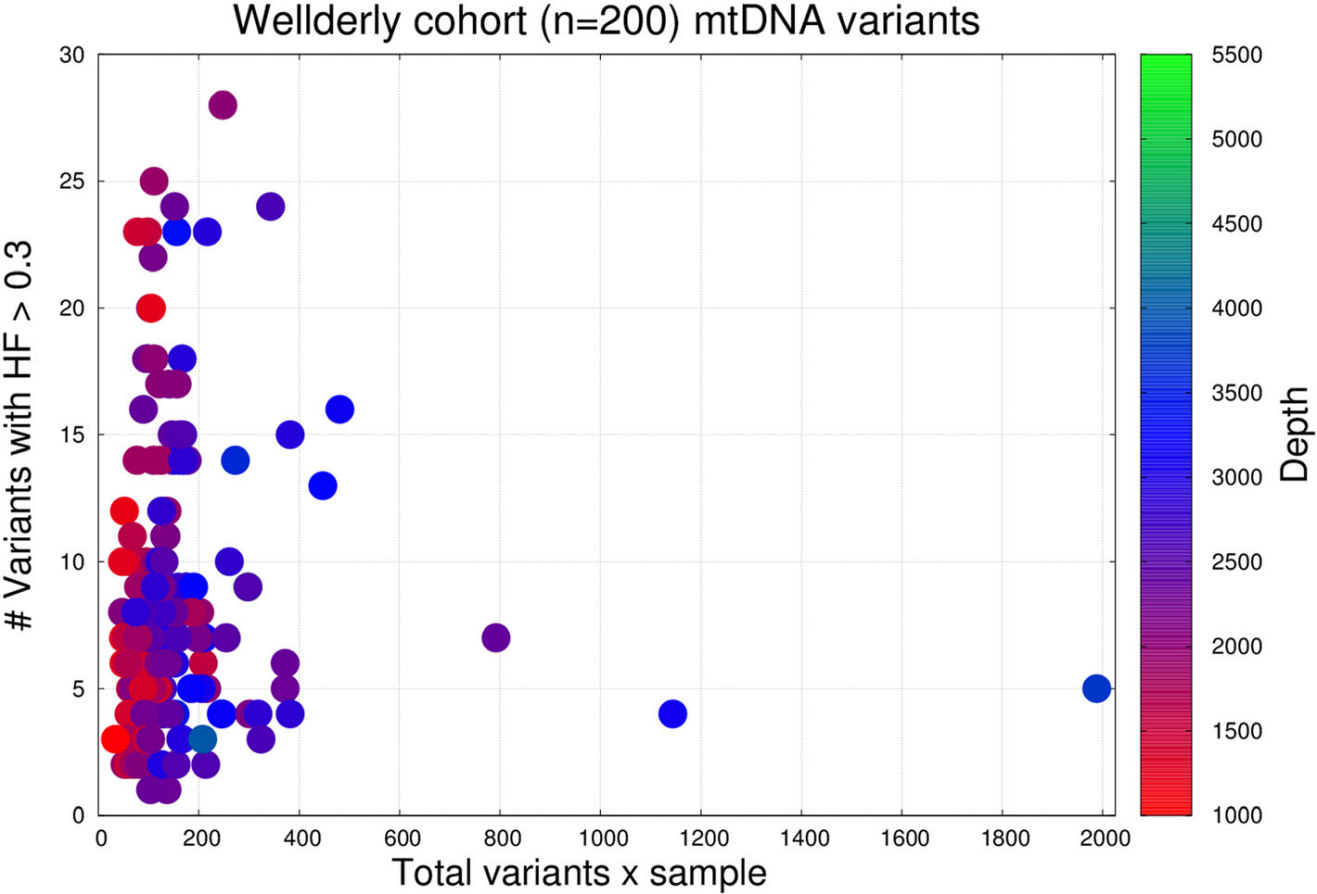
Scatter plot showing the total number of mitochondrial DNA (mtDNA) variants per sample vs. the number of variants with heteroplasmic fraction (HF) > 0.3 in 200 Wellderly individuals. Each dot represents 1 individual. The colouring scheme represents the average (per sample) depth of coverage for mtDNA variants

First, we investigated the effect of coverage (i.e., number of reads per position) on the number of detected mtDNA variants. The DNA for all 200 individuals was extracted from peripheral blood and the average depth (per position) after the sequencing was 2281 ± 594 reads per sample (min value: 1037, max value: 5166). The coverage showed remarkable variability, despite the fact that all samples were sequenced under similar conditions. It is worth mentioning that the disparity in coverage did not stem from differences in the amount of DNA loaded in the plate (see Additional file 1: Figure S1), nor it is correlated to the age of the individuals (see Additional file 1: Figure S2). It is unknown whether this change in DNA abundance is due to actual differences in the number of chromosomal copies, or if it is due to other sample preparation issues during the sequencing process. In any case, even with coverages that exceed 1000X, a common concern is recognizing to what extent the depth affects the capacity to capture essential variants. Figure 1 shows a scatterplot of the total number of variants with respect to the variants having a heteroplasmic fraction > 0.3 (see discussion about the threshold selection at Additional file 1: Figure S3 and [41, 42]). With the exception of three samples, all others consisted of < 500 total variants, the majority having < 400 (median number of variants per individual was 116.5, interquartile range: 88–157, min value: 34, max value: 1988). Samples with an average depth > 2500X consistently contained more total detected variants than those with < 2500X, but the majority of these “additional” variants had extremely low heteroplasmic fractions (see Fig. 1), and therefore many of these variants are potentially noise, sequencing errors, or variant calling artifacts [42]. Increasing the depth of coverage to > 1000X did not affect the number of variants detected at HF > 0.3. In other words, a minimum depth of 300 reads for the alternative allele was sufficient to capture all variants considered in this analysis and variations in depth of coverage did not influence our results.

### 2) Distribution of pathogenic variants across the mtDNA genome

The total number of mitochondrial DNA variants found in all 200 individuals was 30,445 (see Additional file 1: Figure S3). From these, 550 (1.8%) were insertions or deletions, the rest being single nucleotide polymorphisms (SNPs). Four thousand nine hundred sixteen out of the 30,445 (16%) were synonymous variants. When filtered by HF > 0.3 the total number of variants was 1654, which yielded a median number of 7 heteroplasmic variants per sample (interquartile range: 5–9, min value: 1, max value: 28).

**Fig. 2:**
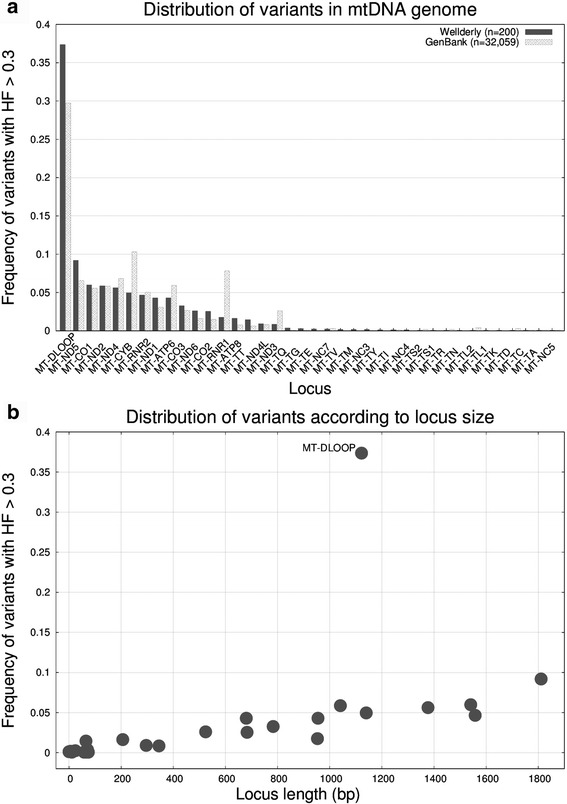
**a** Histogram showing the distribution of the Wellderly cohort heteroplasmic variants (HF > 0.3) across the mitochondrial DNA genome. The loci are numerically sorted according the number of variants. As a reference, we display also values for the GenBank cohort. **b** Scatter plot of the locus length vs. the frequency of heteroplasmic mutations at the locus. Note that the number of mutations correlates almost linearly with the locus size, except for the *MT-DLOOP*

To investigate the distribution of heteroplasmic variants across the mitochondrial genome, we grouped heteroplasmic variants according to their locus and built a histogram with their frequencies (see Fig. 2a). For comparison purposes, we also included the results obtained with 32,059 samples from GenBank (25 June 2016 version) downloaded from the Mitomap database [43, 44]. Despite the difference in cohort size, ancestry, sequencing technology, etc. the amount of heteroplasmic variation per locus seems to be stable in both cohorts. The only exceptions to this rule were the genes *MT-CYB* (mitochondrially encoded cytochrome b) and *MT-RNR1* (mitochondrially encoded 12S RNA), both accumulating a larger number of variants in GenBank cohort [45]. Most heteroplasmic variants tend to accumulate in the *MT-DLOOP* [46]*,* followed by the *MT-ND[X]* complexes (mitochondrially encoded NADHs complexes). The *MT-DLOOP* is the longest noncoding region in vertebrate mtDNA and contains the H-strand replication origin. Two *MT-DLOOP* regions (hypervariable regions HVR1 and HVR2) are known for accumulating more variants than anywhere else in the mitochondrial genome. The *MT-ND[X]* regions are the largest coding loci in the mtDNA genome and, thus, an excess of the absolute number of mitochondrial mutations is expected. On the other hand, tRNA genes are small and should accumulate fewer mutations in total. This is indeed what we observed (see Fig. 2b) with the exception of *MT-DLOOP* region.

After checking variants at the locus level, we sought to investigate specific variants associated with disease in the literature. For this purpose, we selected all Wellderly variants with HF > 0.3 that had been associated with disease in the MitoMap database, and compared their abundance with respect to the GenBank cohort, as well as to that in 1000 Genomes (1000G) cohort [47] (see Table 1). Again, despite the heterogeneity of the data, we found overall concordance. GenBank had spikes in three particular positions, in 3010A (rs3928306) a SNP for which we could not determine anything out of the ordinary other than association studies to eye diseases, and in 11467G and 12372A, both being the only synonymous substitutions found in the list. The total number of pathogenic variants for the Wellderly cohort was 79 (0.39 per sample), whereas for the GenBank was 39,688 (1.24 per sample). When we excluded the 3 variants with large excess (see above) we ended up with 76 (0.38 per sample) vs. 25,910 (0.81 per sample). This two fold increase in the GenBank cohort might be due to differences in the level of heteroplasmy reported, intrinsic errors of GenBank data [45], or enrichment of diseased individuals in the GenBank database. In terms of locus distribution, most of the pathogenic mutations (59%) fell in the *MT-DLOOP* [46] and a very few in the tRNA genes (except for *MT-TE* and *MT-TT*).

**Table 1:** Heteroplasmic variants (HF > 0.3) present in the Wellderly cohort associated with disease in the MitoMap database

Variant	Locus	Aa Change	MitoMap Associated Disease(s)	Wellderly	*n* = 200	GB	*n* = 32,059	1000G
				AC	AF	AC	AF	AF
961C	*MT-RNR1*		DEAF possibly LVNC-associated	2	0.010	317	0.010	0.0066
961G	*MT-RNR1*		Possibly DEAF-associated	2	0.010	123	0.004	0.0008
2352C	*MT-RNR2*		Possibly LVNC-associated	1	0.005	839	0.026	0.0737
2361A	*MT-RNR2*		Possibly LVNC-associated	1	0.005	107	0.003	0.0058
3010A	*MT-RNR2*		Cyclic Vomiting Syndrome with Migraine	1	0.005	5046	0.157	0.1043
3796G	*MT-ND1*	T164A	Adult-Onset Dystonia	1	0.005	174	0.005	0.0029
4454C	*MT-TM*		Possible contributor to mito dysfunction / HTA	1	0.005	185	0.006	0.0041
5913A	*MT-CO1*	D4N	Prostate Cancer/hypertension	2	0.010	241	0.008	0.0029
6253C	*MT-CO1*	M117T	Prostate Cancer	2	0.010	355	0.011	0.0103
6261A	*MT-CO1*	A120T	Prostate Cancer / LHON	1	0.005	176	0.005	0.0054
6489A	*MT-CO1*	L196I	Therapy-Resistant Epilepsy	2	0.010	67	0.002	0.0008
7041A	*MT-CO1*	V380I	Prostate Cancer	1	0.005	5	0.000	0.0004
8393T	*MT-ATP8*	P10S	Reversible brain pseudoatrophy	3	0.015	127	0.004	0.0021
9055A	*MT-ATP6*	A177T	PD protective factor	2	0.010	1581	0.049	0.0189
10454C	*MT-TR*		DEAF helper mut	1	0.005	118	0.004	0.0033
11467G	*MT-ND4*	syn	Altered brain pH	1	0.005	4213	0.131	0.0717
12372A	*MT-ND5*	syn	Altered brain pH	1	0.005	4519	0.141	0.0829
14687G	*MT-TE*		Mito myopathy w respiratory failure	4	0.020	211	0.007	0.0074
15927A	*MT-TT*		Multiple Sclerosis / DEAF1555 inc. penetrance	3	0.015	317	0.010	0.007
16093C	*MT-DLOOP*		Cyclic Vomiting Syndrome	14	0.070	3982	0.124	0.0567
16176T	*MT-DLOOP*		Cyclic Vomiting Syndrome with Migraine	2	0.010	523	0.016	0.0029
16183C	*MT-DLOOP*		Melanoma patients	12	0.060	9632	0.300	0.0869
16192T	*MT-DLOOP*		Melanoma patients	11	0.055	3087	0.096	0.0488
16270T	*MT-DLOOP*		Melanoma patients	8	0.040	3111	0.097	0.0562

As a reference, we also display GenBank and 1000G cohorts. Note that SG-ADVISER mtDNA uses RSRS (Reconstructed Sapiens Reference Sequence) numbering schema whereas data in Mitomap uses Cambridge Reference Sequence (rCRS). We made sure that the numbering schema was equivalent for the variants studied. Acronyms: GB (GenBank), 1000G (1000 Genomes), AF (allele frequency), AC (allele count)

### 3) Phenotypic effects contributing to high heteroplasmic levels

**Fig. 3:**
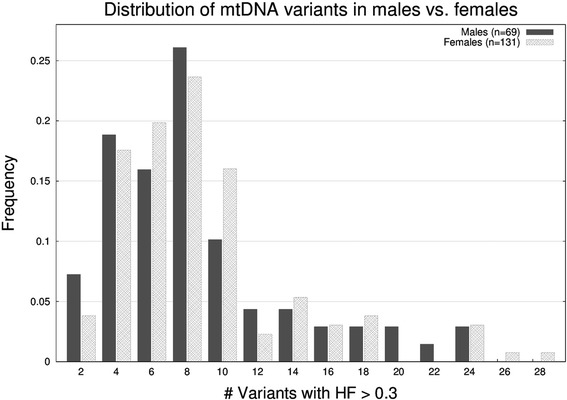
Histogram of the distribution of mitochondrial DNA heteroplasmic variants (HF > 0.3) according to gender in 200 Wellderly individuals

**Fig. 4:**
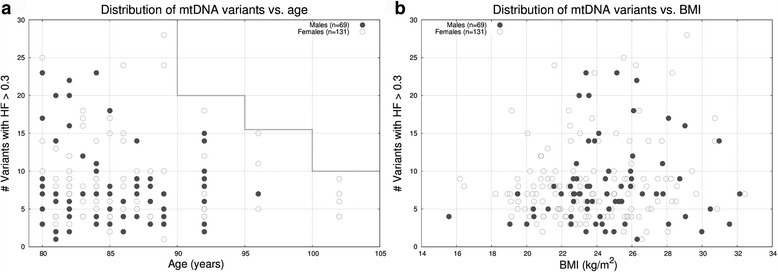
Scatter plots of two phenotypic parameters vs. the number of mitochondrial DNA variants with heteroplasmic fraction (HF) > 0.3 in the Wellderly cohort: **a** age vs. number of variants; note the decrease in number after the age of 90. **b** Body Mass Index (BMI) vs. number of variants, no relation found

Finally, we investigated the amount of heteroplasmy per sample versus several self-reported parameters/conditions such as age, body mass index, smoking status, etc. In Fig. 3 we show a side-by-side histogram of the frequency of variants with HF > 0.3 for males (*n* = 69) and females (*n* = 131). Both genders behave similarly in terms of variant distribution, hence, gender does not appear to be affecting the amount of heteroplasmic variants. In Fig. 4a we compared the number of heteroplasmic variants with respect to age for females and males, and we also did not observed that aging caused an increase in the number of heteroplasmic variants [37] nor is there an interaction between aging and gender on the rate of heteroplasmic variants. Instead, and contrary to our intuition, we observed that individuals over the age of ~90 tended to have a lower number of heteroplasmic variants. By inspecting the 30 variants present in the 5 women older than 100 years old, we observed that 25 (83%) were SNPs, that 13 (43.3%) were synonymous, that only 1 variant (3%) was associated with disease (m.15077G>A, DEAF: maternally inherited nonsyndromic hearing loss) in the Mitomap database, that 1/3 (33%) of the variants were rare (allele frequency < 0.05 in 1000G), that the HF were high (81% had HF > 0.8), and that 16 (53%) were in the *MT-DLOOP* region (87%, 37%, 11%, 18%, 83% and 37% respectively when taking into account the 200 individuals). In fact, a moderate linear relationship (uphill) was observed between the number of heteroplasmic variants in the *MT-DLOOP* and the total number of variants (see Additional file 1: Figure S4), which suggests that the *MT-DLOOP* integrity might play a role in, or be a surrogate for, the overall mutational rate. Taking the above observations together, we hypothesize that inheriting a “stable” mtDNA genome might provide an optimum metabolic efficiency that, as a result, contributes to disease prevention. Based on our data, we cannot determine whether the tendency to have a lower number of heteroplasmic variants after age ~90 is due to sample size, decrease of cell division rate/metabolism associated with age, or if it is due to protective genetic mechanisms. For nuclear DNA, somatic mutations in the context of clonal hematopoiesis have been show to increase with age [48] but recently it has been show that the amount of somatic mutation on induced pluripotent stem cells (iPSC) decreases after age 90 [49].

Lastly, for all other parameters we reported median values, interquartile ranges and *p*-values from a Mann–Whitney U test (see Table 2) used to test for association with heteroplasmic levels. After the Bonferroni correction, we did not observe any association between the number of heteroplasmic variants and any of the parameters studied. The spreadsheet consisting of all the phenotypic information that we compiled is available as Additional file 2: Table S1.

**Table 2:** Median values, [interquartile ranges (sample size)] and Mann-Whitney *U* test *p*-values (uncorrected and Bonferroni corrected (BF)) to test for association between 14 self-reported conditions and the number heteroplasmic variants (HF > 0.3) present in the Wellderly cohort

	Yes	No	Mann-Whitney *U* test	Mann-Whitney *U* test
Condition	Median [IQR (n)]	Median [IQR (n)]	*p*-value	*p*-value (BF)
Blad_control	7.0 [5–8 (6)]	7.0 [5–10 (192)]	0.5974	1.0000
Bph	7.0 [3–9 (24)]	7.0 [5–9 (174)]	0.7815	1.0000
Copd_asthma	6.0 [3–9 (7)]	7.0 [5–9 (193)]	0.4505	1.0000
Depr_anx	7.0 [3–9 (12)]	7.0 [5–9 (188)]	0.5750	1.0000
Dyslipidemia	8.0 [6–12 (46)]	7.0 [4–9 (154)]	0.0833	1.0000
Gerd	7.0 [6–9 (19)]	7.0 [5–9 (181)]	0.4879	1.0000
Glaucoma	7.0 [4–11 (30)]	7.0 [5–9 (170)]	0.8320	1.0000
Hrt	8.5 [7–10 (12)]	7.0 [5–9 (188)]	0.0682	0.9549
Hyperten	7.5 [5–10 (80)]	7.0 [4–9 (120)]	0.0399	0.5585
Hypothyroid	7.0 [5–10 (39)]	7.0 [5–9 (161)]	0.7091	1.0000
Macular_degen	7.0 [5–9 (15)]	7.0 [5–9 (185)]	0.9132	1.0000
Osteoarth	7.0 [5–10 (87)]	7.0 [5–9 (113)]	0.3987	1.0000
Smoking_hist	7.0 [4–9 (89)]	7.0 [5–10 (111)]	0.1922	1.0000
Take_meds	7.0 [5–9 (161)]	7.0 [5–9 (39)]	0.7356	1.0000

Labels: Blad_control: Bladder control problems; Bph: Benign prostatic hyperplasia; Depr_anx: Depression or anxiety; Gerd: Gastroesophageal reflux disease; Hrt: Hormone replacement therapy, Hyperten: Hypertension; Macular_degen: Macular degeneration; Osteoarth: Osteoarthritis; Smoking_hist: Smoking history; Take_meds: Currently taking medications

## Conclusions

We have developed a web tool named SG-ADVISER mtDNA that allows for efficient variant calling, annotation and priorization of variants from human mtDNA SAM/BAM files. The web server has been tested with 200 unpublished mtDNA genomes from a healthy aging cohort and the data has been made public available here. The distribution of heteroplasmic variants in the Wellderly cohort did not substantially differ from that in GenBank or 1000G cohorts. Pending replication, we observed that individuals over the age of ~90 tend to have a low number of heteroplasmic variants in their mitochondrial genomes.

## Additional files

Additional file 1: **Figures S1, S2, S3, S4** and **Text T1**. (DOCX 773 kb)

Additional file 2: A spreadsheet consisting of all the phenotypic information for the 200 Wellderly individuals. (XLSX 86 kb)

## Abbreviations

1000G: 1000 Genomes
BAM: Binary SAM format
CPU: Central processing unit
DEAF: Maternally inherited nonsyndromic hearing loss
DNA: Deoxyribonucleic acid
GUI: Graphical user interface
HDD: Hard disk drive
HF: Heteroplasmic fraction
HTML: Hypertext markup language
HVR: Hypervariable region
mtDNA: Mitochondrial DNA
NGS: Next generation sequencing
PBS: Portable batch system
RAM: Random access memory
RSRS: Reconstructed sapiens reference sequence
SAM: Sequence alignment/map format
SNP: Single nucleotide polymorphism
tRNA: Transfer ribonucleic acid
VCF: Variant call format
WES: Whole exome sequencing
WGS: Whole genome sequencing
