# Supplementary material for: SG-ADVISER mtDNA: a web server for mitochondrial DNA annotation with data from 200 samples of a healthy aging cohort
Source: BMC Bioinformatics. 2017 Aug 18;18:373. doi: 10.1186/s12859-017-1778-6 (PMC5563004; doi:10.1186/s12859-017-1778-6)
Supplement: Supplementary file 1 — Figures S1, S2, S3, S4 and Text T1. (DOCX 773 kb) [file 12859_2017_1778_MOESM1_ESM.docx]

**SUPPORTING FIGURES S1, S2, S3 and S4 and TEXT T1**


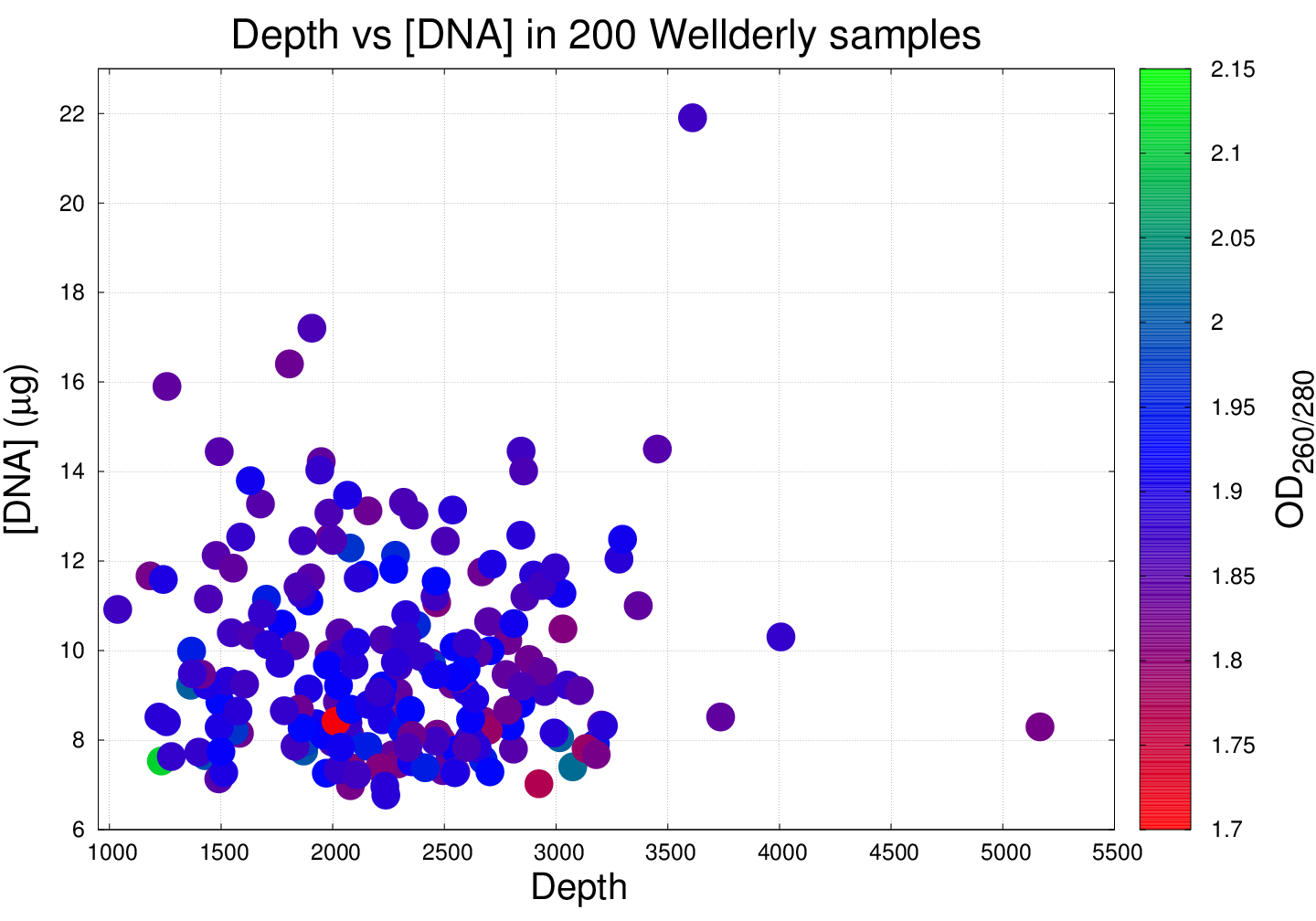


**Supporting Figure S1**. Scatter plot of the average (per position) sample depth vs. the DNA concentration (as loaded in the plate) coloured according the optical density of the ratio of the absorbance at 260 and 280 nm for 200 Wellderly samples. Note that average depth does not seem to be related to the initial amount of DNA present in the sample nor the purity of the DNA.


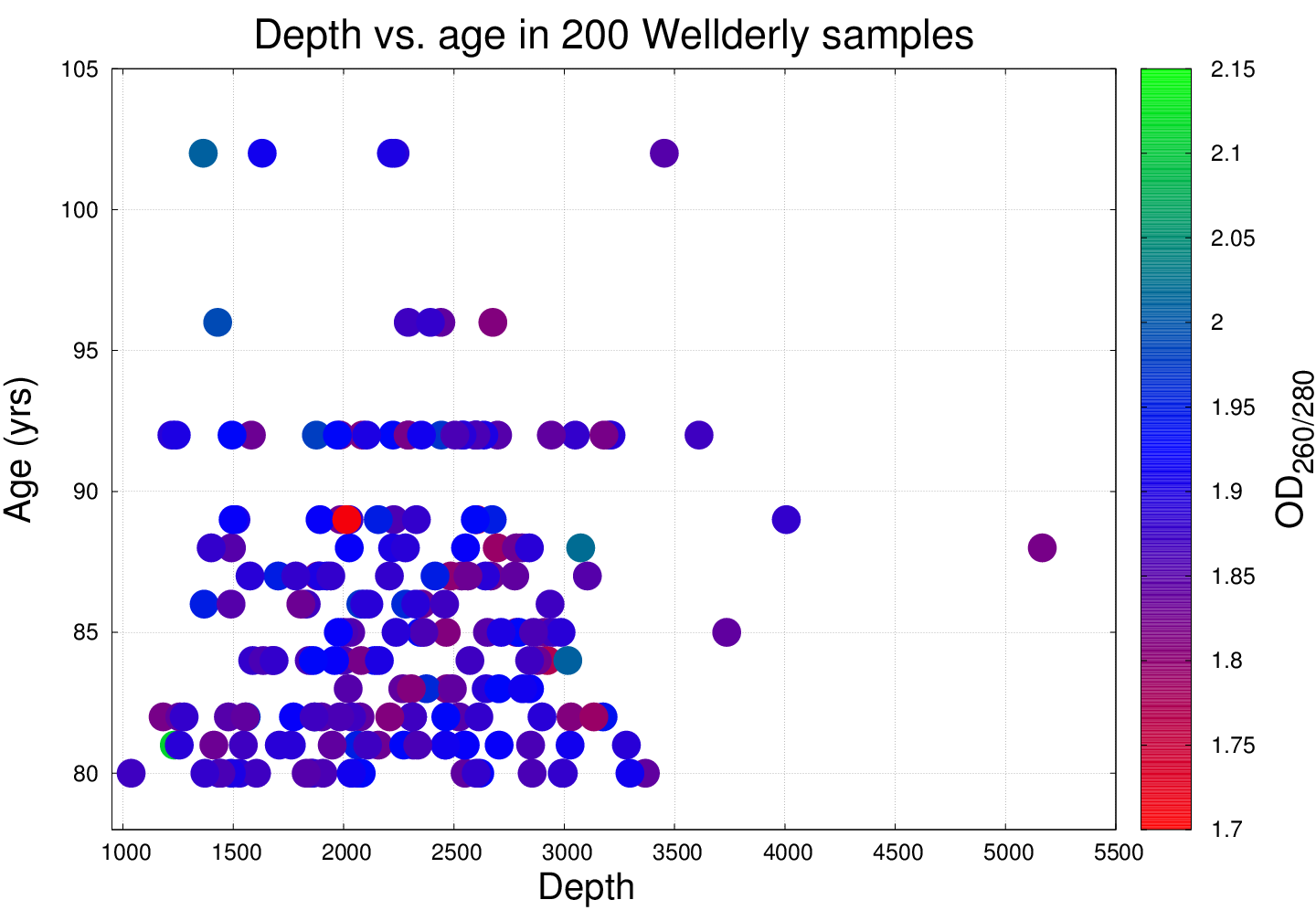


**Supporting Figure S2**. Scatter plot of the average (per position) depth vs. age of the participants, coloured according the optical density of the ratio of the absorbance at 260 and 280 nm for 200 Wellderly samples. No clear correlation was found.

**
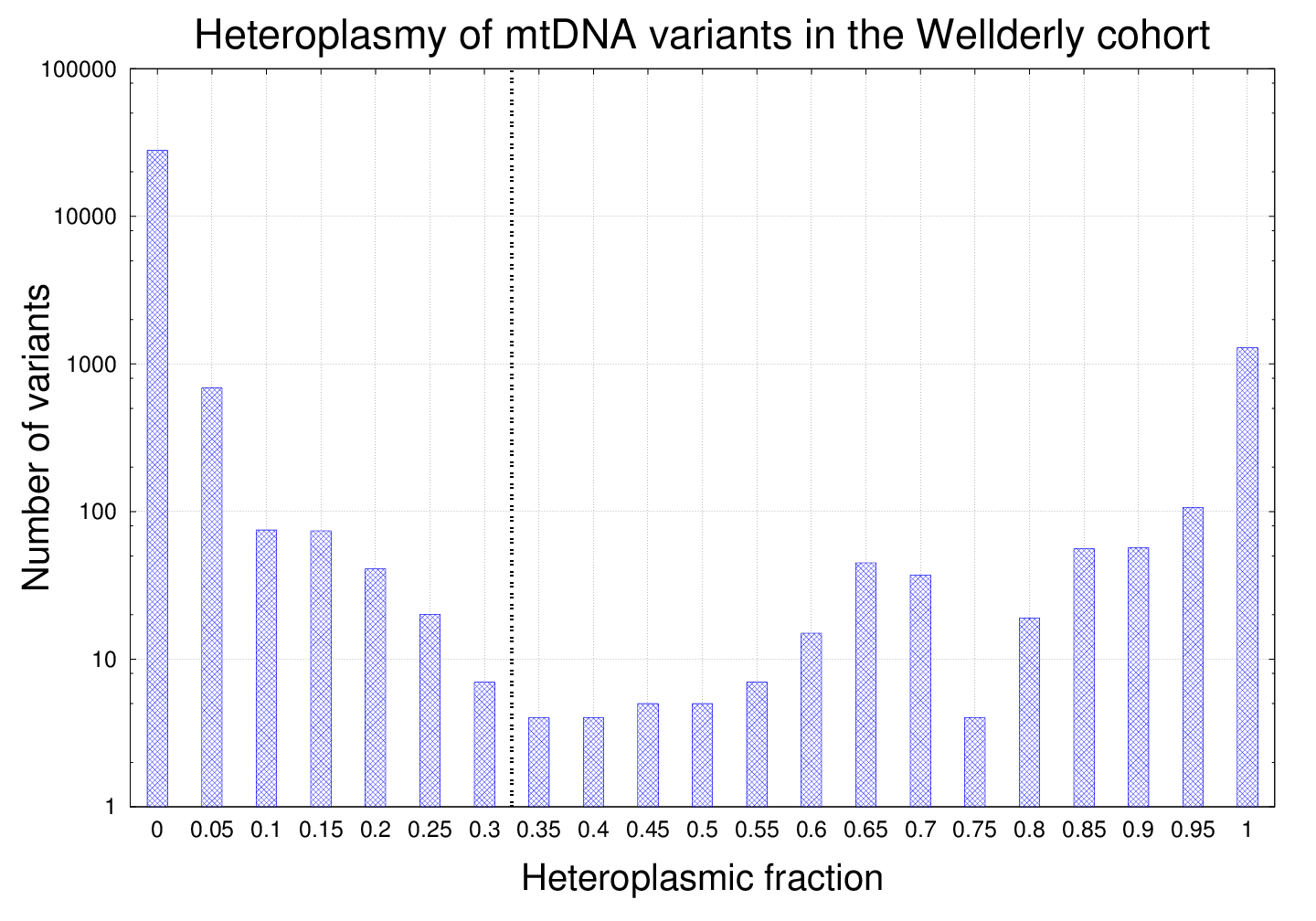
**

**Supporting Figure S3**. Histogram showing the distribution of the all 30,445 mitochondrial DNA (mtDNA) variants according to their heteroplasmic fraction (HF) in the Wellderly cohort (n=200). Note that the y-axis has been transformed to log_10_ scale for visualization purposes. The majority of the variants had extremely low heteroplasmic fractions (HF < 0.05), thus, in order to consider a variant heteroplasmic we established an *ad hoc* threshold of 0.3 (the alternative allele has to have > 30% of the reads; note that variants with HF close to 1.0 (quasi-homoplasmies) are included). In the event that multiple alternative alleles were available, we chose the one with highest heteroplasmic fraction. The reason for establishing a HF threshold is due to the fact that phenotypic effects are only apparent above a certain mutational load, which can be tissue specific. For instance, a variant with HF ~30% in peripheral blood (mesodermal origin) might have a HF > 60% in muscle (endoderm).


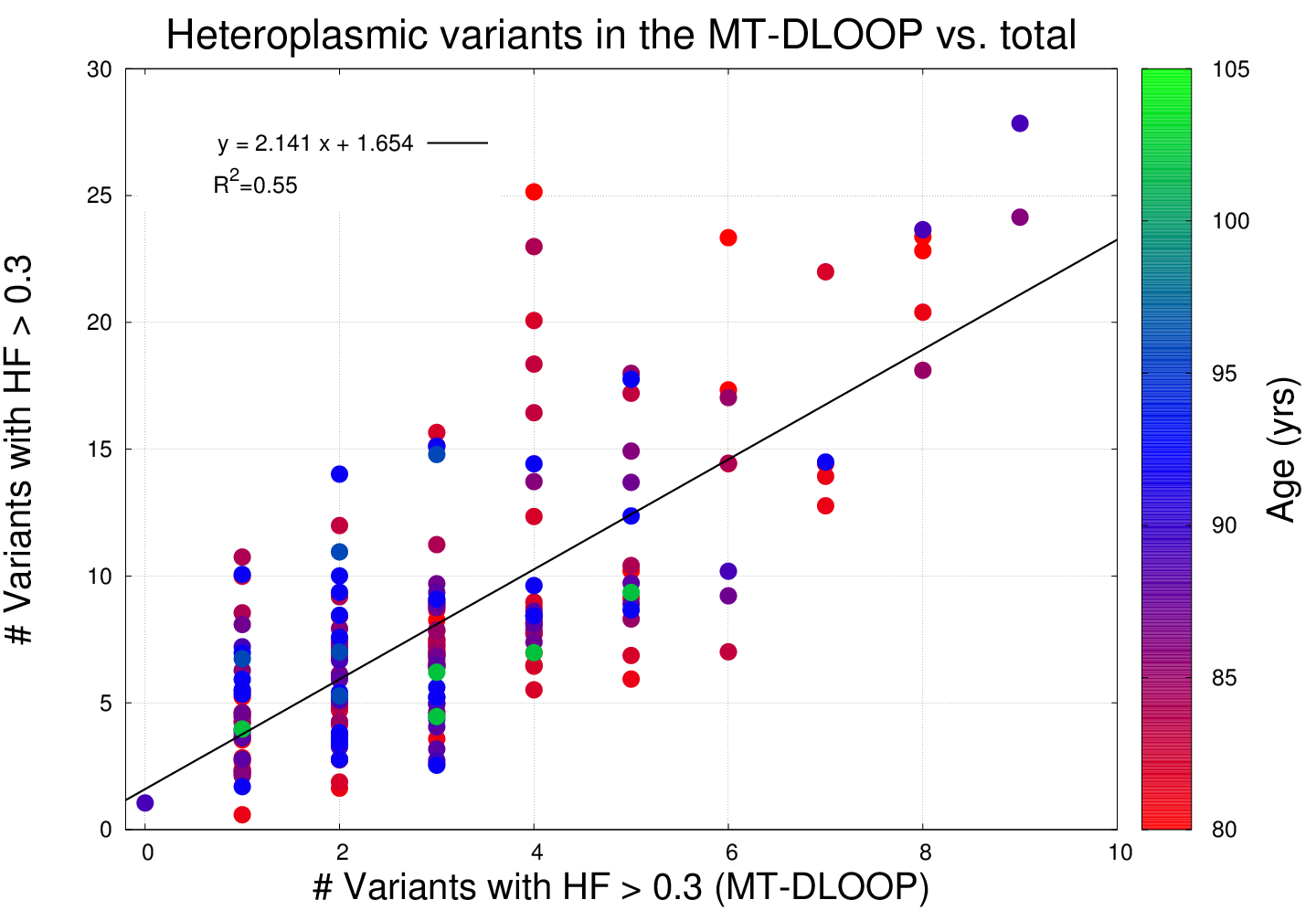


**Supporting Figure S4**. Scatter plot of the number of heteroplasmic variants in the *MT-DLOOP* locus vs. the total number of heteroplasmic variants per sample, coloured by age. Note that 4 out 5 of the females over 100 years old fell into the bottom-left quadrants. For visualization purposes, we introduced random noise (we added a random number between -0.5 and 0.5) in the Y coordinates of each point to provide a better visual separation (otherwise many dots will be overlapped). The linear regression was done without the random noise.

#### Supporting Text T1

SG-ADVISER mtDNA is built on top of [MToolbox v.1.0](https://github.com/mitoNGS/MToolBox) and consists of the following steps:

1. **Preprocessing of SAM / BAM files**

The first thing we do is converting SAM / BAM files into fastq files via *Sam2Fastq.jar* ([PicardTools suite)](https://broadinstitute.github.io/picard/).

1. **Alignment of the reads to a reference genome**

Then we use *mapExome.py* to map the reads onto RSRS (Reconstructed Sapiens Reference Sequence, PMID: 22482806) reference genome. The reads are aligned via [GSNAP](http://research-pub.gene.com/gmap/) (GSNAP 2015-12-31.v7).

1. **Variant calling and annotation**

Variant calling and annotation is performed through MToolBox v1.0. MToolbox is a bioinformatics pipeline to reconstruct and analyze human mitochondrial DNA from high throughput sequencing data. MToolBox performs haplogroup assignment and and prioritization analysis of mitochondrial variants. MToolBox also provides pathogenicity scores, profiles of genome variability and disease-associations for mitochondrial variants. These are the scripts used:

- - SortSam.jar, MarkDuplicates.jar, SamFormatConverter.jar ([PicardTools suite](http://picard.sourceforge.net/)) for SAM / BAM manipulation at several stages of the pipeline and elimination of PCR duplicates.
  - assembleMTgenome.py to assemble the mitochondrial genome and perform variant calling and heteroplasmy quantification, by invoking:
    - mpileup ([SAMtools](http://samtools.sourceforge.net/))
    - mtVariantCaller.py module;
    - VCFoutput.py to report variants in the VCF file (version 4.0). It invokes the vcf module ([PyVCF](https://github.com/jamescasbon/PyVCF) release 0.60);
    - mt-classifier.py for haplogroup prediction;
    - variants_functional_annotation.py for functional annotation;
    - prioritization.py for variant prioritization;
    - summary.py to report statistics about coverage of reconstructed genomes, predicted haplogroups, number of homoplasmic, heteroplasmic and prioritized variants.

1. **Data display**

SG-ADVISER mtDNA transforms the output into a format (JSON) that is rendered in the form of HTML tables.
